# Impact of in-Hospital Left Ventricular Ejection Fraction Recovery on Long-Term Outcomes in Patients Who Underwent Impella Support for HR PCI or Cardiogenic Shock: A Sub-Analysis from the IMP-IT Registry

**DOI:** 10.3390/jpm13050826

**Published:** 2023-05-13

**Authors:** Mario Iannaccone, Luca Franchin, Francesco Burzotta, Giulia Botti, Vittorio Pazzanese, Carlo Briguori, Carlo Trani, Tommaso Piva, Federico De Marco, Giulia Masiero, Maurizio Di Biasi, Paolo Pagnotta, Gavino Casu, Anna Mara Scandroglio, Giuseppe Tarantini, Alaide Chieffo

**Affiliations:** 1 Department of Cardiology, San Giovanni Bosco Hospital, 10100 Turin, Italy; 2 Institute of Cardiology, Fondazione Policlinico Universitario A. Gemelli IRCCS, Università Cattolica del Sacro Cuore, 00100 Rome, Italy; 3 Interventional Cardiology Unit, IRCCS San Raffaele Scientific Institute, 20100 Milan, Italy; 4 Cardiac Intensive Care Unit, IRCCS San Raffaele Scientific Institute, 20100 Milan, Italy; 5 Interventional Cardiology Unit, Mediterranea Cardiocentro, 80100 Naples, Italy; 6 Center for Exercise Science and Sport, Department of Neuroscience and Rehabilitation, University of Ferrara, 44121 Ferrara, Italy; 7 Valvular and Structural Heart Cardiology, Centro Cardiologico Monzino, 20100 Milan, Italy; 8 Department of Cardiac, Thoracic and Vascular Science, University of Padua, 35100 Padua, Italy; 9 Interventional Cardiology Unit, Ospedale Luigi Sacco, 20100 Milan, Italy; 10 Cardiovascular Department, Humanitas Research Hospital, 20089 Rozzano, Italy; 11 Clinical and Interventional Cardiology, Sassari University Hospital, 07100 Sassari, Italy; 12 Advanced Heart Failure and Mechanical Circulatory Support Program, San Raffaele Scientific Institute, Vita Salute University, 20100 Milan, Italy

**Keywords:** Impella, left ventricle support, cardiogenic shock, protect PCI

## Abstract

(1) Background: Percutaneous left ventricle assist devices (pLVADs) demonstrated an improvement in mid-term clinical outcomes in selected patients with severely depressed left ventricular ejection fraction (LVEF) undergoing percutaneous coronary interventions. However, the prognostic impact of in-hospital LVEF recovery is unclear. Accordingly, the present sub-analysis aims to evaluate the impact of LVEF recovery in both cardiogenic shock (CS) and high-risk percutaneous coronary intervention (HR PCI) supported with pLVADs in the IMP-IT registry. (2) Methods: A total of 279 patients (116 patients in CS and 163 patients in HR PCI) treated with Impella 2.5 or CP in the IMP-IT registry were included in this analysis, after excluding those who died while in the hospital or with missing data on LVEF recovery. The primary study objective was a composite of all-cause death, rehospitalisation for heart failure, left ventricle assist device (LVAD) implantation, or heart transplantation (HT), overall referred to as the major adverse cardiac events (MACE) at 1 year. The study aimed to evaluate the impact of in-hospital LVEF recovery on the primary study objective in patients treated with Impella for HR PCI and CS, respectively. (3) Results: The mean in-hospital change in LVEF was 10 ± 1% (*p* < 0.001) in the CS cohort and 3 ± 7% (*p* < 0.001) in the HR PCI group, achieved by 44% and 40% of patients, respectively. In the CS group, patients with less than 10% in-hospital LVEF recovery experienced higher rates of MACE at 1 year of follow-up (FU) (51% vs. 21%, HR 3.8, CI 1.7–8.4, *p* < 0.01). After multivariate analysis, LVEF recovery was the main independent protective factor for MACE at FU (HR 0.23, CI 0.08–0.64, *p* = 0.02). In the HR PCI group, LVEF recovery (>3%) was not associated with lower MACE at multivariable analysis (HR 0.73, CI 0.31–1.72, *p* = 0.17). Conversely, the completeness of revascularisation was found to be a protective factor for MACE (HR 0.11, CI 0.02–0.62, *p* = 0.02) (4) Conclusions: Significant LVEF recovery was associated with improved outcomes in CS patients treated with PCI during mechanical circulatory support with Impella, whereas complete revascularisation showed a significant clinical relevance in HR PCI.

## Bullet Points

1. In the IMP-IT registry, the mean in-hospital change in LVEF was 10.3 ± 1.2%, achieved by 43.6% of CS patients, whereas the mean improvement in HR PCI patients was 3.0 ± 7.2%.
2. In patients presenting with CS, in-hospital LVEF recovery was associated with lower all-cause death, cardiac death, and heart failure rehospitalisation.
3. In patients undergoing HR PCI, LVEF recovery was associated with reduced all-cause death and cardiac death at univariate analysis, but the association with MACE at clinical follow-up was not significant at multivariate analysis; conversely, complete revascularisation was the main independent predictor of events in this group.

## 1. Introduction

Percutaneous left ventricle assist devices (pLVADs) have been increasingly used to unload the left ventricle while maintaining systemic and coronary perfusion in cardiogenic shock (CS) and to provide adequate hemodynamic support during the high-risk percutaneous coronary intervention (HR PCI). The available devices include an intra-aortic balloon pump (IABP), Impella, and venoarterial-extracorporeal membrane oxygenation (VA-ECMO). Unlike long-term LVADs, they are placed percutaneously and provide support from a few hours up to a maximum of 1 month [1]. Although data from the literature report no significant difference in terms of short-term mortality when comparing the pLVAD to the intra-aortic balloon pump (IABP) in the setting of cardiogenic shock [2], a recent meta-analysis suggested that survival may improve in patients supported with Impella, especially when early placement of mechanical circulatory support is guaranteed [3]. In the high-risk PCI scenario, the PROTECT I and II (A Prospective, Randomized Clinical Trial of Hemodynamic Support With Impella 2.5 Versus Intra-Aortic Balloon Pump in Patients Undergoing High-Risk Percutaneous Coronary Intervention) randomized controlled trials (RCTs) evaluated the effectiveness of hemodynamic support in HR PCI and did not report a reduction of major adverse cardiovascular events (MACE) at 30 days [4,5]. Nevertheless, the PROTECT III study, a prospective, single-arm, post-approval study for Impella 2.5 and Impella CP in HR PCI, brought more insight into this field. Accordingly, a comparative analysis recently demonstrated an improvement in clinical outcomes and left ventricle ejection fraction (LVEF) recovery in patients undergoing HR PCI with contemporary practice [6]. Previous studies, especially in the context of chronic coronary syndromes and left ventricular dysfunction (LVD), demonstrated that the increase of LVEF after surgical coronary revascularisation guided by viability state [7] was not associated with improved survival. While LVEF recovery as a marker of better clinical outcomes has been questioned in this scenario, RCTs are often challenging. Therefore, analyses from large multicentre registries such as the IMP-IT [8] offer the opportunity to obtain more insight into the risk–benefit trade-off [9,10,11]. Accordingly, the aim of this IMP-IT sub-analysis is to evaluate the impact on outcomes of in-hospital LVEF recovery in both CS and HR PCI.

## 2. Methods

The IMP-IT study is an investigator-initiated, multicentre, retrospective, national registry study promoted by the Italian Society of Interventional Cardiology (Società Italiana di Cardiologia Interventistica–GISE) [8]. Consecutive patients treated with Impella 2.5, Impella CP, Impella 5.0, and Impella RP, both for CS and HR PCI, in 17 Italian centres were included (IRCCS San Raffaele Scientific Institute, Milan, Italy; Institute of Cardiology, Fondazione Policlinico Universitario A. Gemelli IRCCS, Università Cattolica del Sacro Cuore, Rome; Interventional Cardiology Unit, Mediterranea Cardiocentro, Naples; Interventional Cardiology Unit, Ospedali Riuniti di Ancona, Ancona; Department of Cardiac, Thoracic and Vascular Science, University of Padua Department of Clinical and Interventional Cardiology; IRCCS Policlinico San Donato, Milan; Interventional Cardiology Unit, Ospedale Luigi Sacco, Milan; Cardiovascular Department, Humanitas Research Hospital, Rozzano; Interventional Cardiology Unit, Ospedale San Francesco, Nuoro; Interventional Cardiology, Ospedale San Giovanni Bosco, Turin; Interventional Cardiology Unit, Ospedale di Conegliano; Interventional Cardiology Unit, Azienda Ospedaliera di Perugia; Interventional Cardiology Unit, Vito Fazzi Hospital, Lecce; SS Emodinamica Interventistica, AAS5, Ospedale di Pordenone; Interventional Cardiology Unit, A.O. Bianchi Melacrino Morelli, Reggio Calabria; Interventional Cardiology Unit, Ospedale SS Annunziata, Sassari; Interventional Cardiology Unit, Mestre General Hospital, Mestre). Details regarding included centres and collection of records are described elsewhere [8]. The inclusion criteria for CS and HR PCI populations regarding the devices used in the study as well as the study methodology were previously described [12]. In brief, data related to medical history, procedural characteristics, 30-day- and 1-year outcomes were collected from each centre and included in a pre-specified structured data set. Clinical follow-up data were collected by in-person visits, telephone interviews, and medical notes from any hospital admission or outpatient visits. Adverse events were then adjudicated by two independent cardiologists using source documents provided by each centre. PCI was performed according to each centre’s standard clinical practice. The collection of data at each participating site was performed according to the policies of the local institutional review board/ethics committee. The devices included in this study were the Impella 2.5, Impella CP, Impella 5.0, and Impella RP.

**Figure 1:**
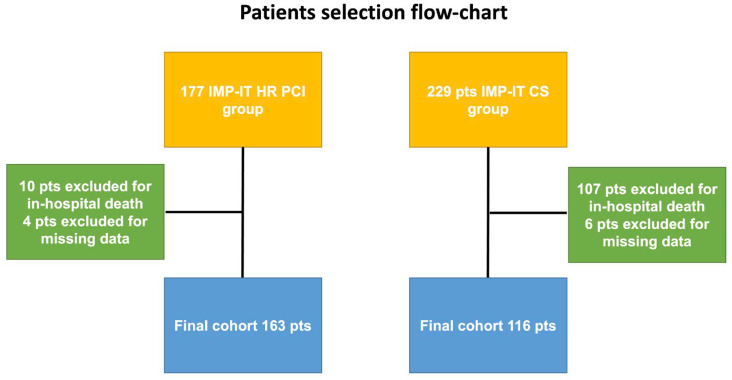
Patient selection flow-chart.

Due to the purpose of this study, the patients who suffered in-hospital death (see Figure 1) were excluded from the analysis. LVEF recovery in each sub-population was defined according to the mean change (an increase of more than 10% of LVEF from pre-PCI to discharge for CS and to more than 3% for HR PCI) as reported in the results section. 

## 3. Study Definition

*Cardiogenic shock.* Criteria for CS included a systolic blood pressure of less than 90 mmHg for longer than 30 min or the use of catecholamine therapy to maintain a systolic pressure of at least 90 mmHg, clinical signs of pulmonary congestion, and signs of impaired organ perfusion with at least one of the following manifestations: altered mental status, cold and clammy skin and limbs, oliguria with a urine output of less than 30 mL per hour, or an arterial lactate level of more than 2.0 mmol per litre.

*High-risk PCI.* HR PCI was defined according to the presence of at least of one clinical and one anatomical high-risk criterion as defined below. High-risk clinical characteristics and comorbidities were defined as: advanced age (>75 years), diabetes mellitus, heart failure with left ventricular ejection fraction <35%, acute coronary syndromes, previous cardiac surgery, peripheral vascular disease, advanced chronic kidney disease (glomerular filtration rate <30 mL/min/1.73 m^2^), chronic obstructive pulmonary disease, concomitant severe aortic valvulopathy, or severe mitral regurgitation. The complexity of coronary anatomies/lesions included: unprotected left main disease, degenerated saphenous vein grafts, severely calcified lesions with the need for rotational atherectomy, last patent conduit, and chronic total occlusions in patients with multivessel disease.

## 4. Devices

The Impella 2.5 device (Abiomed) is a 12 Fr micro-axial pump mounted on a 9 Fr catheter. It is inserted through the femoral artery using a modified Seldinger technique. The pump is advanced retrogradely across the aortic valve into the left ventricle; fluoroscopy guidance is usually used. The Impella 2.5 generates up to 2.5 L/min of flow in the ascending aorta. An activated thrombin time of 160–180 s during pump support is usually recommended for both devices. From 2012, the Impella CP device also became available: it is able to generate up to 4.0 L/min and requires a 14 Fr percutaneous vascular access. The Impella 5.0 device requires a surgical 21 Fr access and is able to generate up to 5.0 L/min. The Impella RP is a right ventricular assist device: it requires a 23 Fr percutaneous femoral vein access and is advanced into the right atrium, across the tricuspid and pulmonic valves, and into the pulmonary artery. It delivers blood from the inlet area, which sits in the inferior vena cava, through the cannula to the outlet opening near the tip of the catheter in the pulmonary artery; it is able to generate up to 4.0 L/min. Selection of each device and support level depends on the clinical scenario, preload status, body size, disease severity, and presence of peripheral artery disease.

## 5. Study Objective

The study aimed to evaluate the impact of in-hospital LVEF recovery on long-term outcomes in patients treated with Impella for HR PCI and CS. The two clinical indications (CS and HR PCI) were analysed separately. 

The primary endpoint of the IMP-IT study was the composite of all-cause death, rehospitalisation for heart failure, left ventricle assist device implantation, or heart transplantation (HT), overall referred to as major adverse cardiac events (MACE) at 1 year. In addition, in-hospital mortality, bleeding events, device-related complications, the occurrence of sepsis, acute kidney injury (AKI), and the need for escalation therapy (defined as the need for ECMO, other left ventricular assist device implantation, or heart transplant) were also evaluated. The extent of revascularisation was graded using a revascularisation index (RI; range: 0 to 1) calculated as follows: [pre-PCI British Cardiovascular Intervention Society Jeopardy Score (BCIS-JS)—post-PCI BCIS-JS)/pre-PCI BCIS-JS]. In this IMP-IT sub-analysis, patients who died while in the hospital were excluded.

## 6. Statistical Methods

Categorical variables are reported as counts and percentages, whereas continuous variables are reported as mean and standard deviation or median and interquartile range (IQR). Gaussian or non-Gaussian distribution was evaluated with the Kolmogorov–Smirnov test. The t-test was used to assess differences between normally distributed continuous variables, paired or unpaired according to the tested variable, the Mann–Whitney U test for non-Gaussian variables, the χ2 test for categorical variables, and the Fisher exact test for 2 × 2 tables. 

We compared the LVEF before the index procedure and at discharge among patients undergoing non-emergent HR PCI and who suffered CS, including those with acute myocardial infarction cardiogenic shock (AMICS) treated with PCI. The comparison was performed using a paired sample t-test. The mean change of EF, which was calculated separately in HR PCI and CS subpopulations, was used as a cut-off for sensitivity analysis between patients with and without EF recovery.

The statistical significance level was set at *p* < 0.05. Forward stepwise multivariable Cox regression analysis, censored at the first event accounting for MACE or at the latest available follow-up, was used to identify clinical and procedural predictors of MACE. Variables with significance < 0.10 were selected to move forward in the final model (variables included in the model for the CS analysis were history of heart failure, diabetes, current smoking status, revascularisation index, and delta EF > 10%, whereas the use of Impella CP, mechanical ventilation, history of prior CABG, revascularisation index, and delta EF > 3% were used in the model for HR PCI).

Concordance probability, which is defined as the probability that predictions and outcomes are concordant, was calculated for each model and reported with Harrell’s C concordance coefficient. All the variables included in the models had less than 5% missing data. Analyses were performed with SPSS^®^ Statistics v24 and STATA v17 (StataCorp, College Station, Texas).

## 7. Results

### 7.1. Cardiogenic Shock Group

#### 7.1.1. Baseline Characteristics

**Figure 2:**
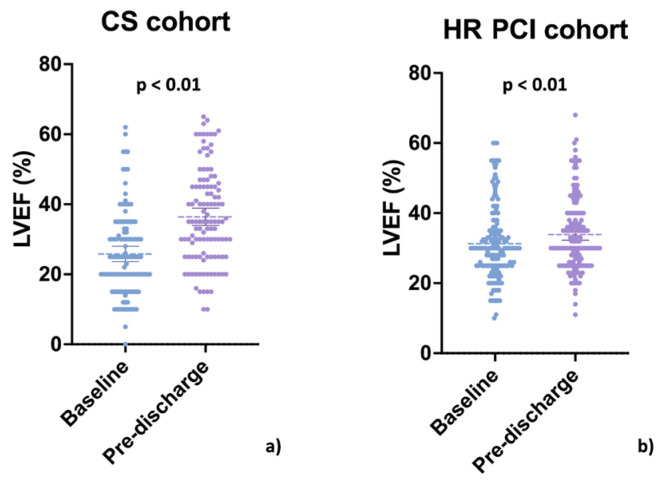
LVEF distribution pre- and post-revascularisation in the cardiogenic shock group (Panel (**a**)) and the high-risk PCI group (Panel (**b**)).

Out of 406 patients included in the IMP-IT registry, 229 (56.4%) presented with or developed CS, and 122 (53.3%) survived at discharge. Echocardiography data were not available in 6 patients (4.9%); therefore, the final cohort included 116 patients (see Figure 1). The mean change in LVEF was 10.3 ± 1.2% *p* < 0.001, with mean pre-PCI and pre-discharge LVEFs of 26.1 ± 12.2% and 36.4 ± 13.5%, respectively (see Figure 2, Panel a). A total of 53 (43.6%) patients achieved a 10% or greater improvement in LVEF. As reported in Table 1, patients who recovered less than 10% LVEF were older, with similar cardiovascular risk factors. On the other side, they had a more frequent history of heart failure, chronic kidney disease, and left circumflex lesions (see Table 1). Patients were supported mainly with Impella 2.5 in both groups (55.4% and 64.3%, respectively). The post-revascularisation BCIS-JS and RI were similar between groups.

#### 7.1.2. Outcomes 

**Figure 3:**
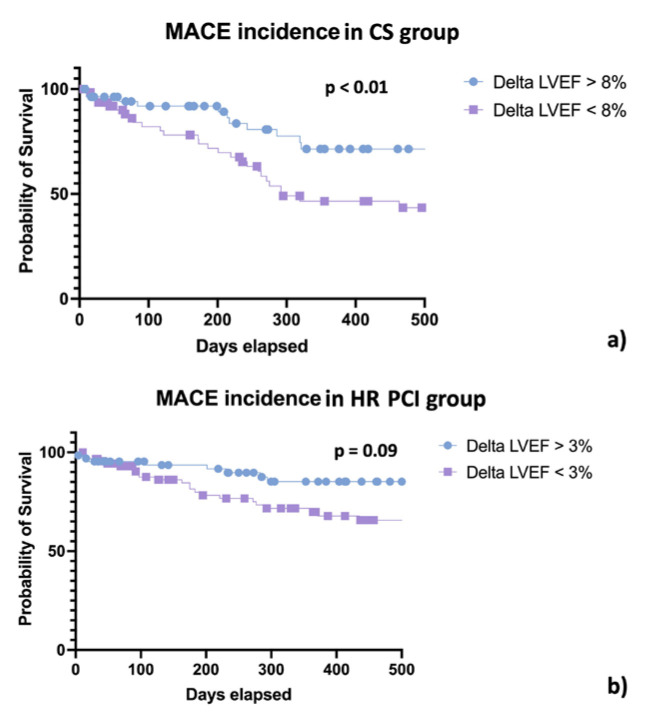
Kaplan–Meier curves for MACE in the cardiogenic shock group (Panel (**a**)) and high-risk PCI group (Panel (**b**)).

Patients who experienced less than a 10% improvement in LVEF at a mean follow-up (FU) of 475 (IQR 67–590) days had a higher incidence of all-cause death, cardiac death, HF hospitalisation, long-term ventricular assistance or transplantation, and MACE (see Table 2 and Figure 3, Panel a). At multiple regression analysis, LVEF recovery of more than 10% (HR 0.23, CI 0.08–0.64, *p* = 0.02) was the strongest predictor for the primary study objective, while the completeness of revascularisation was not. 

### 7.2. High-Risk PCI Group

#### 7.2.1. Baseline Characteristics

**Figure 4:**
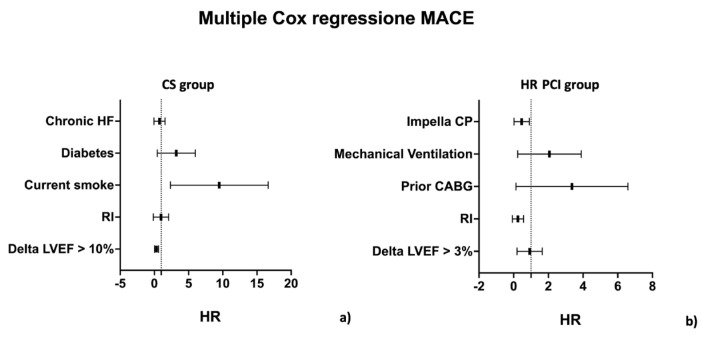
Multiple Cox regression for MACE in the CS group (Panel (**a**)) and HR PCI group (Panel (**b**)).

The IMP-IT registry enrolled 177 patients in the HR PCI group, of which 167 survived at 1 month. Echocardiography data were not available in 4 patients (2.4%), so the final cohort included 163 patients (see Figure 1). The mean change in LVEF was 3.7 ± 7.2 *p* < 0.001- with pre-PCI and pre-discharge mean LVEF of 30.8 ± 10.2% and 33.4 ± 10.2%, respectively (see Figure 2, Panel b). A total of 64 patients (39.7%) achieved a 3% or greater improvement in LVEF. Patients who did not recover at least 3% in LVEF were older, with similar cardiovascular risk factors. Conversely, they were more frequently female and affected by chronic kidney disease (see Table 3). Patients were supported mainly with Impella 2.5, and in both groups post-revascularisation, BCIS-JS and RI were similar (see Figure 4, Panel a).

#### 7.2.2. Outcomes

At a mean FU of 574 (IQR 57–740) days, the population that achieved greater LVEF recovery experienced a lower incidence of all-cause death and cardiac death, whereas heart failure hospitalisations, LVAD, or transplantation tended to be consistent with previously reported outcomes (*p* = 0.09) (see Table 4 and Figure 3, Panel b).

At multiple regression analysis, only the completeness of revascularisation was independently associated with MACE (HR 0.11, CI 0.02–0.62, *p* = 0.02), whereas the impact of LVEF recovery was not significant (HR 0.73, CI 0.31–1.72, *p* = 0.17, see Figure 4, Panel b). 

## 8. Discussion

The main findings of this real-world analysis based on the IMP-IT registry can be summarised as follows:

a. In the CS group, half of the patients experienced an improvement in LVEF of at least 10%. Conversely, an increase of only 3% was achieved by about 40% of patients in the HR PCI group.
b. In patients presenting with CS, in-hospital LVEF recovery was the main predictor of lower all-cause death, cardiac death, HF hospitalisation, and MACE at clinical follow-up. Instead, complete revascularisation, but no improvement in EF, was the main predictor of adverse events in patients undergoing HR PCI.

According to the latest European Society of Cardiology guidelines on Acute and Chronic Heart Failure [13], short-term mechanical circulatory support in AMICS is recommended with class IIa indication, despite the lack of solid RCTs data (level of evidence C), as most of the evidence is derived from observational studies and registries. IABP has failed to improve outcomes in this setting [14]. PVADs such as Impella have been developed to improve the prognosis of these patients; they offer advantages over IABPs, including an increase in cardiac output and afterload reduction [15]. In the present analysis, we found that a significant improvement in LVEF in the CS group was associated with lower mortality and morbidity. 

It is worth noting that an improvement in LVEF of more than 10%, rather than the completeness of revascularisation, was associated with a reduction of MACE at multivariable analysis; this finding allows us to speculate that LVEF recovery might be among the main determinants of better prognosis in the setting of cardiogenic shock. Regarding the extent of revascularisation, while the use of Impella may reduce the risk associated with complete revascularisation in comparison to culprit-only revascularisation, as reported in the AMICS registry [16], its additive role in multivessel PCI is inconsistent with the findings of the CULPRIT-SHOCK (PCI Strategies in Patients with Acute Myocardial Infarction and Cardiogenic Shock) trial [17]. A recent paper published by the ROMA-VERONA group [18], enrolling 64 patients supported with Impella in the setting of an acute coronary syndrome, showed opposite results; however, only 26.3% of patients presented with cardiogenic shock. Further studies are needed to investigate the optimal interplay between completeness of revascularisation, supported PCI, and outcome in patients with AMICS.

In the high-risk PCI group, patients with severe LVD showed a less significant improvement of LVEF compared to the CS setting, and no correlation with better outcomes. Interestingly, the main predictor of outcome was the extent of complete revascularisation, consistent with published literature [19]. Despite the increasing implementation of pLVADs in HR PCI, the use of these devices might lead to non-negligible complications; therefore, careful management and upfront planning are warranted [20]. Historically, patients with multivessel disease and acceptable surgical risk were referred to surgical revascularisation, according to ESC guidelines which recommend coronary artery bypass graft (CABG) with a Class I indication (level B of evidence). PCI is indicated in one- and two-vessel disease or for three-vessel disease, if the feasibility of complete percutaneous revascularisation is assessed by the Heart Team (class IIa indication, level of evidence C) [21]. 

The latest REVIVED-BCIS2 (Percutaneous Revascularization for Ischemic Left Ventricular Dysfunction) trial failed to prove the clinical benefit of revascularisation with PCI over optimal medical therapy (OMT) [22]. Nevertheless, improvement in quality of life appeared to favour PCI, and spontaneous myocardial infarction and intracardiac defibrillator interventions were also reduced in the PCI arm, despite the significant limitation of this trial [23]. Interestingly, at 12 months FU, the LVEF changed from baseline by 2.0% in the PCI arm, compared to 1.1% in the OMT arm, whereas complete revascularisation was achieved in 71% of patients in the PCI group. The rate of PCI procedures requiring mechanical circulatory support was not reported, as well as data regarding ischemia testing and angiographic patterns of the vessels and lesions [22]. Compared to that of the REVIVED-BCIS2 trial, our population had more extensive and severe coronary artery disease, which required complex intervention and a higher rate of left main revascularisation.

Taken together with these findings, our results might suggest that in patients with chronic coronary artery disease and heart failure, revascularisation may reduce ischemia viewed as a trigger for future MACE; however, the chronic myocardial damage and hibernation of the myocardium is difficult to revert. Even though a short-term improvement in LVEF is not a marker of better prognosis, as suggested by the “Surgical Treatment for Ischaemic Heart Failure” (STICH) trial, other factors may contribute to a better outcome, such as the reduction of arrhythmic burden [24] and of the recurrence of MI [25].

Indeed, the impact of complete revascularisation, especially in the setting of the acute coronary syndrome, the impact of plaque characteristics [26], and the hemodynamic stabilisation that the mechanical support may increase to achieve better intraprocedural results with more aggressive post-dilatations [27] reducing long-term cardiovascular events have to be taken into account.

## 9. Limitations

The present study is a retrospective sub-analysis of an observational registry; therefore, some limitations must be considered, and the results should be interpreted as hypothesis-generating. The scenario of a real-world cohort is invariably associated with inclusion bias but offers the advantage of wide data generalisability. First, event monitoring was not standardised across clinical centres, which could have led to underreporting of adverse events. Second, a general protocol for Impella use in CS was not mandatory; therefore, the timing of Impella placement in the CS population was not standardised. Third, most of the patients were treated with Impella 2.5. Moreover, a core-lab revision of all angiographic procedures, completeness of revascularisation (anatomical versus functional), and echo data to reduce inter- and intra-observer variability were not available, nor was any prespecified operator experience required to enter the registry. 

## 10. Conclusions

In-hospital LVEF recovery was associated with improved outcomes in AMICS treated with PCI during Impella mechanical circulatory support, whereas only complete revascularisation showed a significant clinical relevance in HR PCI. 

## Figures and Tables

**Table 1:** Baseline characteristics of the CS cohort in patients with and without left ventricle ejection fraction recovery.

	EF Recovery < 10% (63 pts)	EF Recovery > 10% (53 pts)	*p*-Value
*Age (mean * *± SD)*	65.5 ± 9.9	59.3 ± 14.7	<0.01
*BMI (mean * *± SD)*	26.1 ± 4.1	25.6 ± 4	0.66
*Female gender (%)*	24.6	26.8	0.81
*Current smoker (%)*	18.8	20.2	0.8
*Hypertension (%)*	62.5	48.8	0.14
*Dyslipidemia (%)*	48.4	32.1	0.91
*NIDDM (%)*	36.9	28.6	0.34
*IDDM (%)*	15.9	14.5	1
*Prior PCI (%)*	36.9	23.2	0.12
*Prior CABG (%)*	4.6	3.6	1
*PAD (%)*	15.4	14.3	1
*Chronic heart failure (%)*	36.9	12.5	<0.01
*CKD (%)*	30.8	14.3	0.05
*STEMI (%)*	58.5	58.9	1
*LAD (%)*	73.2	74.5	1
*LCx (%)*	67.3	36	<0.01
*RCA (%)*	69.6	45.1	0.01
*BCIS-JS pre-PCI (mean * *± SD)*	8.5 ± 5.2	7.7 ± 5.8	0.4
*n. of diseased vessels (mean * *± SD)*	2.2 ± 0.95	1.64 ± 1	0.03
*RI (mean * *± SD)*	0.74 ± 0.35	0.76 ± 0.3	0.26
*BCIS-JS post-PCI (mean * *± SD)*	2.2 ± 2.8	2.3 ± 3.6	0.8
*Lactates (mg/dl) (mean * *± SD)*	4.7 ± 3.7	5.8 ± 4.2	0.22
*Baseline creatinine (mg/dl) (mean * *± SD)*	1.7 ± 1.7	1.2 ± 0.5	0.41
*MAP (mmHg) (mean * *± SD)*	68 ± 14.6	67.1 ± 23.1	0.83
*Impella duration of support (hours) (mean * *± SD)*	85.3 ± 88.9	89.4 ± 68.1	0.82
*Impella CP (%)*	38.5	30.4	0.44
*Impella 2.5 L (%)*	55.4	64.3	0.35
*Impella 5 L (%)*	3.1	0	0.49
*Inotrope use (%)*	69.4	64	0.6
*OHCA (%)*	20	29.1	0.28
*Mechanical ventilation (%)*	62.5	73.2	0.24

BMI: body mass index; NIDDM: non-insulin-dependent diabetes mellitus; IDDM: insulin-dependent diabetes mellitus; PCI: percutaneous coronary intervention; CABG: coronary artery by-pass graft; PAD: peripheral artery disease; HF: heart failure; CKD: chronic kidney disease; STEMI: ST segment elevation myocardial infarction; LM: left main; LAD: left anterior descending; LCx: left circumflex; RCA: right coronary artery; BCIS-JS: British Cardiovascular Intervention Society myocardial jeopardy score; RI: revascularisation index; MAP: mean artery pressure; OHCA: out-of-hospital cardiac arrest.

**Table 2:** Outcomes of cardiogenic shock cohort in patients with and without left ventricle ejection fraction recovery.

	EF Recovery < 10% (63 pts)	EF Recovery > 10% (53 pts)	*p*-Value
*All-Cause death (%)*	27.7	10.9	0.02
*Cardiac death (%)*	23.8	7.3	0.02
*MI (%)*	1	0.8	0.9
*Heart failure hospitalisation (%)*	26.6	9.3	0.02
*LVAD or transplantation (%)*	25	7.4	0.01
*30-day survival (%)*	89.2	98.1	0.07
*MACE (%)*	50.8	21.4	<0.01
*Any device-related complication (%)*	36.9	25.7	1
*Sepsis (%)*	30.8	36.4	0.56
*CIN (%)*	50	42.9	0.42

MI: myocardial infarction; HF: heart failure; LVAD: left ventricle assist device; MACE: major adverse cardiovascular events; CIN: contrast-induced nephropathy.

**Table 3:** Baseline characteristics of the HR PCI cohort in patients with and without LVEF recovery.

	EF Recovery < 3% (99 pts)	EF Recovery > 3% (64 pts)	*p*-Value
*Age (mean ± SD)*	74.5 ± 9.4	70.6 ± 9.2	0.01
*BMI (mean ± SD)*	25.9 ± 3.6	25.8 ± 3.4	0.85
*Female gender (%)*	20	7.4	0.04
*Current smoker (%)*	18	24.2	0.54
*Hypertension (%)*	63	80.3	0.68
*Dyslipidemia (%)*	63	59.1	0.62
*NIDDM (%)*	47	44	0.75
*IDDM (%)*	24.2	23.1	1
*Prior PCI (%)*	26	18.2	0.26
*Prior CABG (%)*	15	13.5	1
*PAD (%)*	27	22.7	0.58
*Chronic heart failure (%)*	54.5	53	0.87
*CKD (%)*	42.4	25.8	0.03
*Baseline creatinine (mg/dl)*	1.4 ± 1.4	1.2 ± 0.8	0.47
*LM (%)*	50.5	47	0.75
*LAD (%)*	93.9	97	0.47
*LCx (%)*	85.6	90.9	0.34
*RCA (%)*	75.3	89.4	0.07
*Use of RA (%)*	30.6	12.3	0.01
*BCIS-JS pre-PCI (mean ± SD)*	11.5 ± 4.2	12.2 ± 3.8	0.29
*n.of diseased vessels (mean ± SD)*	2.2 ± 2.8	2.7 ± 0.7	0.27
*BCIS-JS post-PCI(mean ± SD)*	3.4 ± 2.8	3.1 ± 2.7	0.59
*RI (mean ± SD)*	0.7 ± 0.2	0.74 ± 0.23	0.4
*Impella duration of support (hours) (mean ± SD)*	13.4 ± 42	16.4 ± 52	0.68
*Impella CP (%)*	31	40.9	0.24
*Impella 2.5 L (%)*	69	54.5	0.07
*Impella 5 L (%)*	0	3	0.16
*Inotrope use (%)*	7.4	9.1	0.77
*Mechanical ventilation (%)*	18.4	15.2	0.67

BMI: body mass index; NIDDM: non-insulin-dependent diabetes mellitus; IDDM: insulin-dependent diabetes mellitus; PCI: percutaneous coronary intervention; CABG: coronary artery by-pass graft; PAD: peripheral artery disease; HF: heart failure; CKD: chronic kidney disease; STEMI: ST segment elevation myocardial infarction; LM: left main; LAD: left anterior descending; LCx: left circumflex; RCA: right coronary artery; BCIS-JS: British Cardiovascular Intervention Society myocardial jeopardy score; RI: revascularisation index; MAP: mean artery pressure.

**Table 4:** Outcomes of HR PCI cohort in patients with and without LVEF recovery.

	EF Recovery < 3% (99 pts)	EF Recovery > 3% (46 pts)	*p*-Value
*All-Cause death*	17.6	6.3	0.02
*Cardiac death*	14	4.8	0.04
*MI*	7.7	3.1	0.3
*Heart failure hospitalisation*	11.2	16.1	0.46
*LVAD or transplantation*	0	1.6	0.41
*30-day survival*	89.2	98.1	0.07
*MACE*	26	18.2	0.26
*Any device-related complication*	12	9.1	0.61
*CIN*	24.4	27.8	0.69

MI: myocardial infarction; HF: heart failure; LVAD: left ventricle assist device; MACE: major adverse cardiovascular events; CIN: contrast-induced nephropathy.

## Data Availability

Data is unavailable due to privacy.
